# Serum Concentration of Leptin in Pregnant Adolescents Correlated with Gestational Weight Gain, Postpartum Weight Retention and Newborn Weight/Length

**DOI:** 10.3390/nu9101067

**Published:** 2017-09-27

**Authors:** Reyna Sámano, Hugo Martínez-Rojano, Gabriela Chico-Barba, Estela Godínez-Martínez, Bernarda Sánchez-Jiménez, Diana Montiel-Ojeda, Maricruz Tolentino

**Affiliations:** 1 Departamento de Nutrición y Bioprogramación, Instituto Nacional de Perinatología, Secretaría de Salud, 11000 Ciudad de México, Mexico; ssmr0119@yahoo.com.mx (R.S.); gabyc3@gmail.com (G.C.-B.); eygodinez@hotmail.com (E.G.-M.); cruz_tolentino@yahoo.com.mx (M.T.); 2 Sección de Posgrado e Investigación, Escuela Superior de Medicina del Instituto Politécnico Nacional, 11340 Ciudad de México, Mexico; 3 Coordinación de Medicina Laboral, Instituto de Diagnóstico y Referencia Epidemiológicos (InDRE) “Dr. Manuel Martínez Báez”, Secretaría de Salud, 01480 Ciudad de México, Mexico; 4 Subdirección de Investigación en Intervenciones Comunitarias del Instituto Nacional de Perinatología, Secretaría de Salud, 11000 Ciudad de México, Mexico; emiberna20@yahoo.com.mx; 5 Universidad del Valle de México, Chapultepec, 11810 Ciudad de México, Mexico; lauramont.nutricion@gmail.com

**Keywords:** Leptin, adolescent pregnancy, pregestational body mass index gestational, weight gain, birth weight

## Abstract

**Introduction**: Gestational weight gain is an important modifiable factor known to influence fetal outcomes including birth weight and adiposity. Leptin is normally correlated with adiposity and is also known to increase throughout pregnancy, as the placenta becomes a source of leptin synthesis. Several studies have reported positive correlations between cord blood leptin level and either birthweight or size for gestational age, as well as body mass index (BMI). **Objective**: To determine the correlation of prenatal leptin concentration in pregnant adolescents with their gestational weight gain, postpartum weight retention, and weight/length of their newborn. **Methods**: A cohort study was conducted on pregnant Mexican adolescents from Gestational Week 26–28 to three months postpartum (*n* = 168 mother–child dyads). An anthropometric assessment was made of each pregnant adolescent, and the serum level of leptin and the intake of energy were determined. The newborn was evaluated each month during postpartum. Clinical records were reviewed to obtain sociodemographic data. Bivariate correlations, tests for repeating measurements and logistic regression models were performed. **Results**: Leptin concentration gradually increased during the third trimester of pregnancy. At Gestation Week 36, leptin level correlated with gestational weight gain. When comparing adolescents that had the lowest and highest concentration of leptin, the former presented a mean of 6 kg less in gestational weight gain (inter-subject leptin concentration, *p* = 0.001; inter-subject energy intake, *p* = 0.497). Leptin concentration and gestational weight gain exerted an effect on the weight of the newborn (inter-subject leptin concentration for Week 32, *p* = 0.024; inter-subject gestational weight gain, *p* = 0.011). Newborn length was associated with leptin concentration at Week 28 (leptin effect, *p* = 0.003; effect of gestational weight gain, *p* = 0.722). **Conclusions**: Pregnant adolescents with leptin concentration over 20 ng/mL showed a greater gestational weight gain. Leptin concentration correlated with length and weight of the newborn.

## 1. Introduction

Under normal conditions of feeding cycle, the concentration of leptin reflects the quantity of adipose tissue; therefore, its concentration increases with increasing adiposity [1]. Women have significantly higher leptin concentrations than men and these gender differences seem to be present from birth, when there are no notable differences in body composition or hormonal levels between genders [2].

In addition, pregnant women who are carrying a female fetus have a significantly higher increase in leptin concentration compared to pregnant women who are carrying a male fetus. This evidence indicates a differential resistance to the action of leptin that could have adaptive importance for reproduction [3]. Increased leptin concentrations in pregnant women are unlikely to impose a reduction in energy intake as pregnancy progresses, because during this period it is crucial to maintain a positive energy balance to maintain the demands of energy for fetal development, for breastfeeding and for preserving maternal health. In contrast, it is speculated that increased leptin concentrations increase maternal fat deposits and fat mobilization to increase availability and support the transplacental transfer of lipid substrates to the fetus, particularly after the second trimester of gestation [4]. Thus, pregnancy is an example of the temporary resistance to leptin, which is similar to that of obese individuals [5].

About 17 million women in the world under 19 years of age get pregnant every year [6]. In Mexico, approximately one of five births (18.4%) is by adolescent mothers [7]. Adolescents have not yet stopped growing and developing, and therefore their nutritional requirements are different from adult women. Thus, it is common for pregnant adolescents to either not cover these requirements or exceed them when a doctor recommends a gestational weight gain that corresponds to an adult woman.

This situation puts pregnant adolescents at risk for an excessive or deficient gestational weight gain, which in turn alters fetal growth and development, either causing an excessive degree of adiposity upon birth or retarding intrauterine growth [8,9,10]. It has been reported that malnutrition or overweight and obesity at early ages of life are associated with intrauterine malnutrition and/or chronic diseases such as obesity, diabetes, hypertension and cardiovascular disorders. These diseases manifest themselves with increasing frequency in adult life [11]. Moreover, in adult women, an inadequate diet together with an elevated production of leptin during pregnancy can lead to abnormal gestational weight gain, giving rise to a greater number of complications during pregnancy as well as considerable postpartum retention of gestational weight gain [12].

Many of the physiological changes that occur during pregnancy are controlled by hormones such as leptin [13,14]. Produced by adipose tissue and the placenta, leptin has been shown to perform a pivotal role in fetal growth and development [15]. The accumulation of adipose tissue is known to cause an elevated concentration of leptin, but it is not clear whether the latter is correlated with greater weight gain during adolescent pregnancy.

In adult women, it has been suggested that the maternal weight determines the ratio between the weight of the newborn and the subsequent body mass index (BMI). Furthermore, excessive maternal weight in adult women is related to an increase in the supply of energetic substrates in the mother–child dyad. However, it is unknown whether this mechanism is similar in pregnant adolescents, whose own personal growth and development has not yet come to an end and who in the majority of cases do not suffer from overweight or obesity [16].

It has been demonstrated that the serum concentration of leptin in pregnant women is correlated with their body fat and body weight [17,18]. Consequently, the serum concentration of leptin in adult women may be a predictor of postnatal weight retention, a possibility that has not been well explored in adolescent mothers [16].

Since Mexico is second in worldwide childhood obesity and second in adult obesity, postnatal weight retention has important public health implications for pregnant adolescents and their children. Therefore, identifying adolescents at risk could help to prevent an excessive gestational weight gain and thus avoid obesity at an early age [19].

It is unclear whether the leptin concentration of pregnant adolescents affects the weight and length of their newborns. The aim of the present study was to determine the correlation between the serum concentration of leptin in pregnant adolescents and three factors: gestational weight gain, postnatal weight retention, and the weight and length of the newborn.

## 2. Material and Methods

### 2.1. Study Design

A cohort study was conducted with the participation of the National Institute of Perinatology (Instituto Nacional de Perinatología (INPer)) and the Superior School of Medicine (Escuela Superior de Medicina, Instituto Politécnico Nacional), both located in Mexico City. INPer is a third level institution whose main functions are healthcare and research. Adolescents attended at INPer are from Mexico City and states nearby, they have no social security coverage and are from low–medium socioeconomic status, where the prevalence of teenage pregnancy is high.

The sample included 168 dyads of pregnant adolescents and their newborns. All participants received prenatal control and their children were born in the toco-surgical unit of INPer. The study was conducted between January of 2009 and December of 2016. Sampling was non-probabilistic, based on consecutive cases that complied with certain selection criteria. Participating adolescents were 10–19 years old according to the World Health Organization definition [20], were primiparous, had a normal evolution of the fetus, did not show clinical evidence of chronic, infectious or metabolic disease. The criteria for non-inclusion were consumption of alcohol, tobacco or drugs, pregnancy with twins, or pregnancy as a product of rape. Participants were eliminated if they developed gestational diabetes or preeclampsia. Data were not adjusted by ethnic group because in Mexico City there is very little ethnic diversity. In Mexico, approximately 90% of the people constitute what can be considered one ethnic group and one culture. Within this 90% of the population, skin color is not a factor of social isolation. Cultural diversity and ethnicity exist in Mexico representing about 10% of the population. This segment of the population is comprised of many cultures, maintaining distinct traditions and languages. As they want to maintain their cultural identity, indigenous people rarely look for medical care in the National Institute of Perinatology, but instead in their local communities. Thus, the approximately 4% of the population constituted by indigenous people in the metropolitan area of Mexico City is underrepresented in the sample. The study began as of the end of the second trimester or onset of the third trimester of gestation (26–28 weeks). Follow-ups were scheduled every four weeks until three months postpartum. To avoid lost-to-follow-up, a telephone call reminder was made the day before the scheduled visit. In case it was not possible to attend, the appointment was rescheduled within 24–48 h. Despite this strategy, 31 participants were lost or eliminated during the study for the following causes: 5 gave birth at a different hospital, 6 developed preeclampsia, 2 had gestational diabetes, 12 stopped attending the hospital for personal reasons, and 6 moved and contact with them was lost or they had no one to accompany them to the visits. Approval was received for this study from the Ethics in Research Committee of the National Institute of Perinatology (212250-49451, 29 November 2005), in accordance with the Declaration of Helsinki. Written informed consent was obtained from adolescents who participated in this study, as well as from their parents or guardians. The dataset supporting the conclusions of this article is available upon request.

### 2.2. Data Collection Methodology

Clinical records of the participating adolescents were carefully reviewed to assure that they complied with selection criteria. All eligible candidates were personally invited to participate in the study. Once they accepted, the reason for the study and its methodology were explained. The benefits of participation were pointed out, including free check-ups and medical studies up to three months postpartum, even if they withdrew from the study (for whatever reason). An appointment card was provided to each participant during the first appointment so that they could keep track of their visits.

Age, age of menarche, pregestational weight, occupation, level of education, marital status and socioeconomic level were obtained from clinical records once the adolescents were selected and agreed to participate. After childbirth, the clinical record was again reviewed to obtain the weight, length and gestational age of the newborn, the latter of which was calculated by the methods of Capurro and Ballard.

### 2.3. Anthropometric Assessment

Under standard conditions, the participants were requested to change into a hospital gown before evaluating the anthropometric parameters with the Lohman technique [21], including their weight and height (used to find the BMI by dividing the weight in kg by the height in meters squared). The participant was asked about her weight at least three months before pregnancy. This value and her height were used to calculate pregestational BMI, in accordance with the established procedure of the Institute of Medicine (IOM) of the USA [22]. Gestational weight gain was found by subtracting the pregestational weight from that obtained at Gestation Week 38–40 (maximum weight in kg); then it was classified as adequate, insufficient or excessive according to the IOM recommendations [22,23].

Anthropometric evaluation of the newborn was performed in the first 24 h of life. The weight of the newborn was determined in grams on a pediatric scale (SECA 374, model “Baby and Mommy”; accuracy of 0.010 kg). Newborn length was measured in centimeters with an infant meter (SECA 416; accuracy of 0.1 cm). The weight and length were transformed into a *Z*-score, which was used to identify the classification: −3 = very low, −2 = low, 0 = adequate, 2 = high, 3 = very high [23]. Infants’ weight and height were assessed monthly for the first three months as a follow-up evaluation.

### 2.4. Examination of the Diet

On Gestation Week 28, the diet and quantity of energy intake was explored through the frequency of food consumption questionnaire, proposed by the Food and Agriculture Organization (FAO) of the United Nations. This instrument was previously validated in a Mexican population and the data obtained represented the typical intake [24]. Each of the responses was coded and later entered into a program validated in Mexico [18] to ascertain the approximate energy intake. The energy requirement (Kcal) of each of the participants was calculated with the following formula: 8.5 − (30.8 × age) + 1 × (10 × weight + 934 × height) + 25 + 452 [22]. Subsequently, the percentage of adequacy was calculated, considering a value from 90% to 110% as adequate, a value <89% as low, and a value >110% as excessive [25].

### 2.5. Biochemical Evaluation

During Gestation Weeks 28, 32 and 36, each of the participants was asked to go to the clinic from 7:00 to 8:00 a.m. for a blood sample withdrawal (after having fasted 8–10 h). Following asepsis and antisepsis, a quantity of 5 mL of blood was obtained by venipuncture, employing the Vacutainer vacuum system. Once the blood was coagulated, the sample was centrifuged for 10 min at 3500 rpm to obtain serum, which was stored in different aliquots at −70 °C to await the leptin assay.

### 2.6. Leptin Determination

Leptin concentration in serum was determined by ELISA technique, utilizing a kit (Quantikine**^®^** ELISA Human Leptin Immunoassay, R&D Systems Inc., Minneapolis, MN, USA) for the quantitative measurement of human leptin concentration in serum and plasma and a microplate reader for absorbance (ELISA Bio-Rad, model 680 Benchmark Plus, Bio-Rad, Hercules, CA, USA).

The assay was performed according to manufacturer’s instructions. The range of the assay was between 1.0 and 100 ng/mL. The sensitivity of the procedure was 0.5 ng/mL. The experimental variation and the variation coefficient were 2.3–6.2% and 2.1–5.3%, respectively.

### 2.7. Socioeconomic Evaluation

The socioeconomic level was assessed during the first interview based on a questionnaire of the Mexican Association of Market Survey and Public Opinion (Asociación Mexicana de Investigación de Mercados y Opinión Pública), which consisted of ten questions with a determined score and final category. In this way, the socioeconomic level was assigned to each participant by employing the following scale: A/B, the highest standard of living; C+, above average; C, average; D+, below average; D, low or austere; E, the lowest income and standard of living [26].

### 2.8. Ethical Considerations

The adolescents that agreed to participate in the study, as well as their parents or tutors, signed informed consent during the first interview. The research protocol was approved by the Ethics in Research Committee of the institution.

Collection and analysis of information was confidential, as the data was stored with an identification number. Ethical questions of autonomy, confidentiality and safety were taken into account. Informational flyers were distributed to all participants with nutritional orientation aimed at helping them to achieve the adequate eating habits for pregnant adolescents. All recommendations were in accordance with the official Mexican norms of the Secretary of Health (Norma Oficial Mexicana; NOM-043-SSA2-2005) [27].

### 2.9. Statistical Analysis

For univariate analysis, frequencies, measures of central tendency and dispersion were performed to describe the general variables. A bivariate correlation was performed between the different variables. Tests for repeating measurements were performed to establish the effect over time of the leptin concentration on gestational weight gain as well as the weight and length of the newborn.

Forward stepwise linear regression models were performed using gestational weight gain and weight and length of the newborn as dependent variables, leptin concentration, energy intake, BMI and pregestational maternal height were consider as independent variables. Additionally, means were compared with the Wilcoxon test. Furthermore, the means of leptin concentration were compared to gestational weight gain by using ANOVA test. All data were analyzed in the SPSS statistical program version 21 for Windows (IBM^®^ Corp, North Castle, NY, USA). Statistical significance was considered at *p* < 0.05.

## 3. Results

The general characteristics of the cohort of 168 dyads of pregnant adolescents and their newborns (given medical attention from 2009 to 2016 at INPer, Mexico City) are shown in Table 1. The study began as of Gestation Weeks 26–28 and continued until three months postpartum. The mean chronological age was 15 years old and the mean gynecological age was four years.

Overall, average gestational weight gain was 13 kg. Nevertheless, according to pregestational BMI, low weight category had a median weight gain of 13 kg (p25–p75: 13–17 kg), normal weight classification had 12 kg (9.4–16.4 kg), overweight had 11 kg (8.4–16 kg) and for obesity weight gain was 4 kg (−4–15 kg). The newborns had an average weight of 3038 g and an average length of 49.5 cm.

The majority of the adolescents were housewives, either married or cohabitating, and belonged to a low or very low socioeconomic level. The pregnancy ended in Caesarian section in 50% of the cases. Evaluation of the BMI of participants demonstrated that seven (4%) of the adolescents were underweight, 127 (75%) normal weight, 28 (17%) overweight, and six (4%) obese.

**Figure 1:**
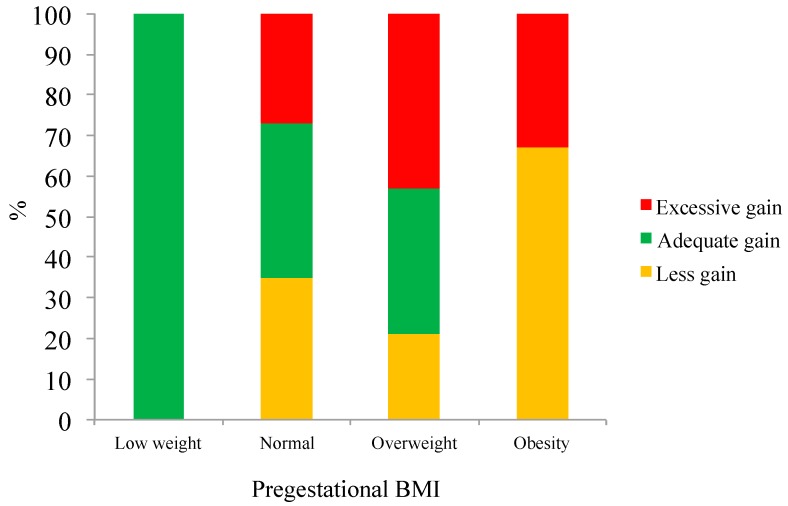
Gestational weight gain (%) according to pregestational BMI.

Figure 1 shows that both overweight and obese adolescents were more likely to exceed the gestational weight gain recommendations.

**Figure 2:**
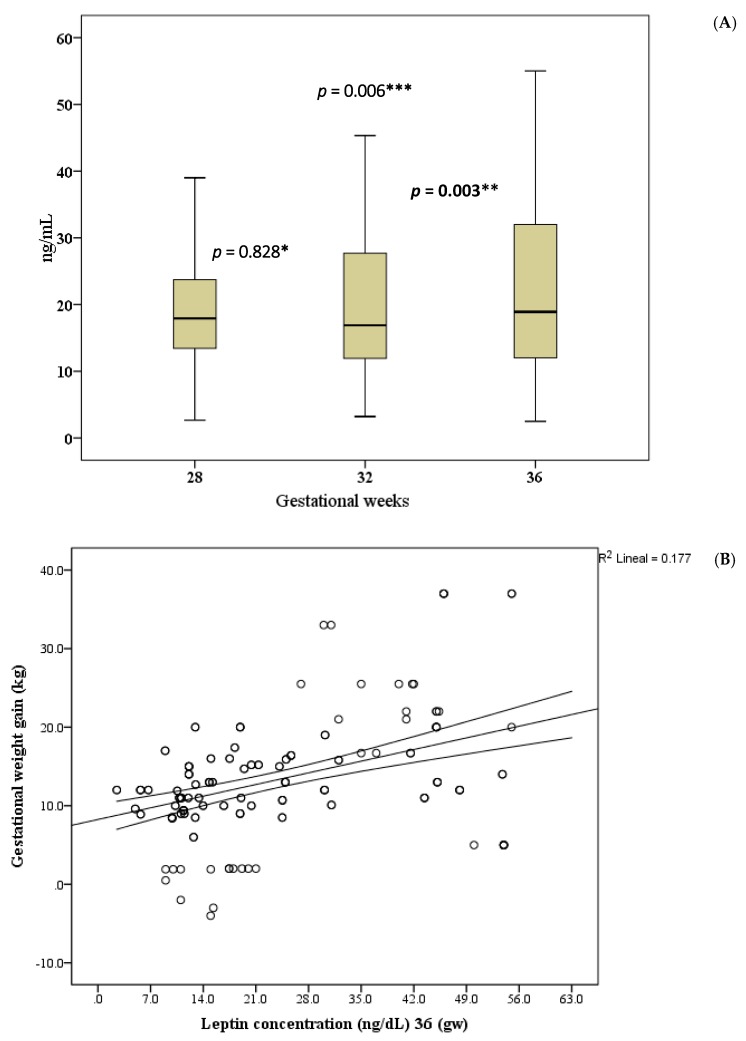
Leptin and gestational weight gain: (**A**) tendency of leptin (ng/mL) from 28 to 36 gestational weeks; (*): Gestation Week 28 and 32; (**): Gestation Week 32 and 36; and, (***): Gestation Week 28 and 36 and (**B**) correlation between gestational weight (kg) and leptin concentration (ng/mL) at Gestation Week 36.

Adolescents initiated their pregnancy with a mean pregestational weight of 51.2 kg, reaching an average maximum gestational weight of 64.4 kg (during Gestation Weeks 38–40). They lost a mean of 9 kg by the end of three months postpartum, resulting in an average weight gain (compared to the pregestational weight) of 4.6 kg. During Gestation Weeks 32–36, there was a significant increase in the serum concentration of leptin. Figure 2A shows that difference between leptin concentrations at Gestation Week 28 and 32 was no statistical significant (*p* = 0.828). However, a statistically significant difference was observed between leptin at gestation week 32 and 36 (*p* = 0.003) and at 28 and 36 week (*p* = 0.006). In addition, a correlation was observed between leptin concentration and gestational weight gain (Figure 2B).

When correlations were performed between leptin concentrations and BMI at Gestation Weeks 28, 32 and 36, Gestational week 36 was the only moment with a significant correlation (*r* = 0.229 *p* = 0.001); meanwhile, there were no significant correlations for Gestation weeks 28 and 32 (*r* = 0.125, *p* = 0.110 and *r* = 0.103, *p* = 0.189, respectively).

Leptin concentration (ng/mL) at gestation weeks 28, 32 and 36 was greater in adolescents giving birth by Caesarian section than by vaginal delivery: 19 (14–26) vs. 17 (11–23), *p* = 0.112; 18 (12–32) vs. 15 (10–23), *p* = 0.008; 24 (13–41) vs. 14 (11–25), *p* = 0.001. Nevertheless, BMI was not associated with type of delivery (*p* = 0.575) using a Chi square Pearson comparison; and when comparing BMI as continuous variable depending of type of delivery using a T-Student test, it was not statistical difference either (*p* = 0.850). Caesarian section was more frequent in adolescents with excessive gestational weight gain (*p* = 0.073). Regarding gender of the newborn, there was no significant difference in the leptin concentration at Gestation Week 28 or 32. However, a gender difference was noted in relation to leptin concentration at Gestation Week 36, being higher (*p* = 0.047) in the group of adolescents that delivered a baby girl (20; range 12–41 ng/mL) than those having a baby boy (18; range 11–24 ng/mL).

**Figure 3:**
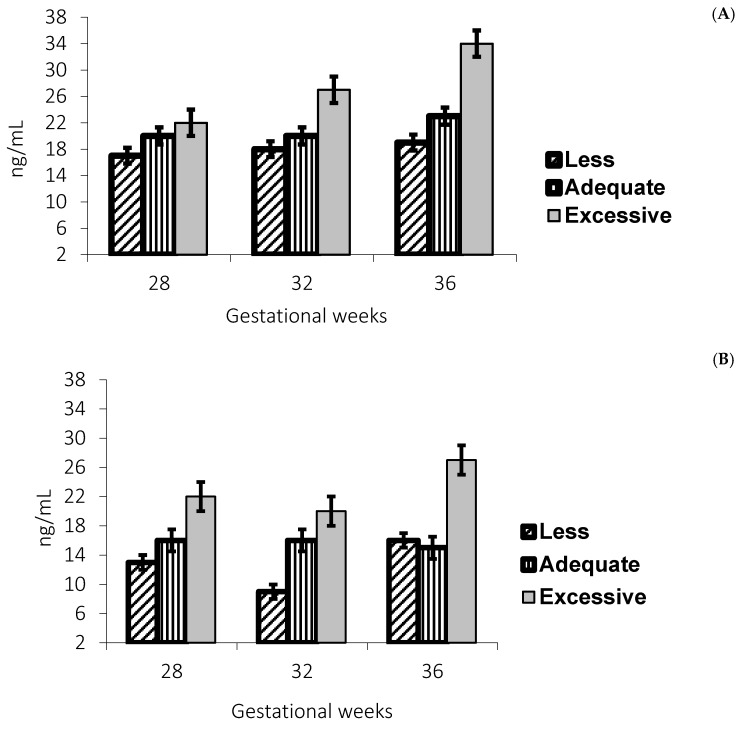
In (**A**), at Gestation Week 28 *p* = 0.027, post hoc less vs. excessive *p* = 0.008; at Gestation Week 32 *p* = 0.001, post hoc less vs. excessive *p* = 0.001, adequate vs. excessive *p* = 0.003; and at Gestation Week 36 *p* = 0.001, post hoc less vs. excessive *p* = 0.001, adequate vs. excessive 0.001. In (**B**), at Gestation Week 28 *p* = 0.001, post hoc less vs. excessive *p* = 0.001, adequate vs. excessive *p* = 0.014; at Gestation Week 32 *p* = 0.004, post hoc less vs. excessive *p* = 0.001, adequate vs. excessive *p* = 0.043; and at Gestation Week 36 *p* = 0.054, post hoc less vs. excessive *p* = 0.057, adequate vs. excessive 0.031. Leptin concentrations (ng/mL) at 28, 32 and gestation week 36, according to gestational weight gain, in adolescents according pregestational BMI: (**A**) low and normal *n* = 134; and (**B**) overweight and obese *n* = 34.

Leptin concentrations were higher in adolescents with excessive gestational weight gain in those with a normal and overweight pregestational BMI, as shown in Figure 3A,B.

Since the greatest leptin concentration was found at Gestation Week 36, the median (20 ng/mL) was used as a reference point to analyze all the adolescents during their pregnancy. Adolescents with a leptin concentration >20 ng/mL at Gestation Week 36 had greater weight at Gestation Week 40 (*p* = 0.002) as well as at 15 (*p* = 0.008), 30 (*p* = 0.036) and 60 (*p* = 0.038) days postpartum. Leptin concentration was analyzed in relation to the BMI, also determined by time (F = 29.659, *p* = 0.001) and leptin concentration at Gestation Week 36 (F = 20.259, *p* = 0.001), with a squared effect.

Regarding the anthropometric parameters of the newborns, a correlation was observed between their weight/length and the maternal serum concentration of leptin at Gestation Weeks 28, 32 and 36, a relation that continued up to the first month postpartum. Nevertheless, no significant difference was detected between the leptin concentration and neonate weight/length when calculating the *Z*-score.

An association was detected between maternal leptin concentration and neonate length from birth to three months of age. Table 2 shows significant correlations in maternal leptin concentration at gestation week 32 with birth weight and at one month of life, while maternal leptin concentrations at 28 and 32 with length in almost all measurements. Pregestational BMI correlated to weight and length at all weeks, at birth and at one month.

Pregestational BMI correlated inversely with gestational weight gain (*r* = −0.263, *p* ≤ 0.001). Gestational weight gain correlated with leptin concentrations at Gestation Week 28 (*r* = 0.327, *p* ≤ 0.001), at Gestation Week 32 (*r* = 0.398, *p* ≤ 0.001) and at Gestation Week 36 (*r* = 0.424, *p* ≤ 0.001).

The analysis of data with a linear regression model revealed that three variables predict 19% of the change in maternal gestational weight: leptin concentration at Gestation Week 36, pregestational weight, and energy intake percentage of adequacy. The weight of the newborn could be predicted by the maternal gestational weight gain and the concentration of leptin at Gestation Week 32 and 36. The variables related to newborn length were the leptin concentration at Gestation Week 32 and maternal height, showing a value of *R*^2^ = 0.085 (Table 3).

There was negative correlation between gestational weight gain and percentage of adequacy for energy intake (*r* = −0.227, *p* = 0.003).

**Figure 4:**
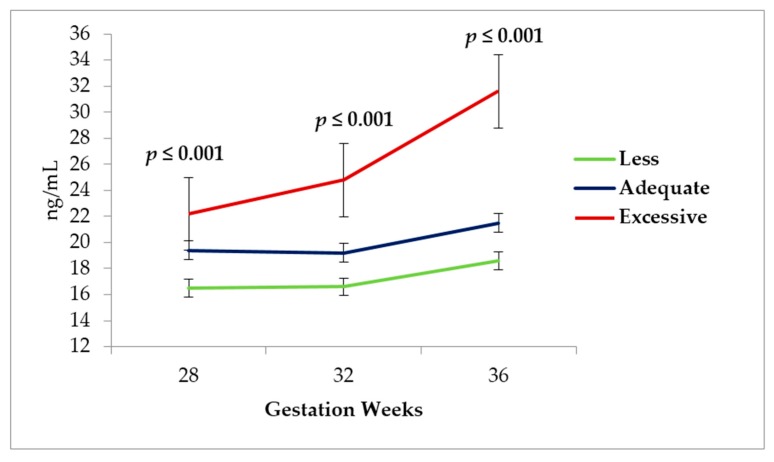
Evolution of serum concentration of maternal leptin (ng/mL) according to gestational weight gain.

According to the analysis of the leptin concentration during the three different measurement times of pregnancy, the highest concentration at Gestation Week 32 and 36 occurred in the adolescents displaying an excessive gestational weight gain (Figure 4).

## 4. Discussion

A significant increase was found in the concentration of leptin during the last trimester of gestation, which could possibly owe itself to the mobilization of the sources of nutrients necessary for fetal growth, such as maternal adipose tissue [16]. In the present study, a leptin concentration above 20 ng/mL during the third trimester of gestation was related to a greater gestational weight gain, which coincides with a study by Lacroix et al. [4] on a group of pregnant adults in Quebec, Canada, who also demonstrated an association between a higher leptin concentration and a greater gestational weight gain. This association suggests that despite peripheral hyperleptinemia, positive energy balance is achieved during pregnancy by a relative decrease in central leptin concentrations and resistance to the effects of leptin on target neuropeptides that regulate energy balance [5].

Contrary to the present results, Kim et al. [28] observed no association between the leptin concentration in the third trimester of gestation and any of the variables of maternal weight in a Korean population. Additionally, gestational weight gain did not correlate with postpartum weight retention. This discrepancy may be due to the reduced number of participants (*n* = 75) and/or the fact that diets of the Korean women and Mexican adolescents are different.

The positive association detected in this study between the leptin concentration at Gestation Week 28 and 36 and the BMI (and consequently gestational weight gain) is similar to what was reported for a group of adult women from New York with a BMI of 19.8 to 26 kg/m^2^. In the latter study, the high concentration of leptin at the beginning of pregnancy was able to predict a greater risk of overweight and obesity in the women that were vulnerable to weight gain (a history of diabetic and obese parents, sedentary, with diets high in carbohydrate intake, etc.) [29]. The present findings extend current knowledge, suggesting that this positive association implies an increased risk of overweight and obesity at a very early age in adolescent mothers having a predisposition to overweight, and in their children as well. Similarly, Stein et al. [29] found that an elevated level of leptin at the onset of the pregnancy of adult women correlated with the development of overweight and obesity. Leptin concentration was the predictive factor for an excess of weight in both adolescent and adult women undergoing pregnancy.

In this study, overweight adolescents showing a high leptin concentration had a more pronounced gestational weight gain than the recommended by the IOM. This positive association could therefore exist in general for overweight pregnant adolescents. Likewise, Lacroix et al. [4] found a stronger correlation between the leptin concentration and gestational weight gain in overweight pregnant adolescents versus those with normal weight. This is worrisome since overweight adolescents, similar to overweight adult women, are more prone to gain excessive weight during gestation than women who are of normal weight or obese.

The current results not only provide evidence that leptin concentration may be related to gestational weight gain of pregnant adolescents, but also to weight and length of their newborns. The latter is in agreement with a report on a group of 42 pregnant adult women with and without obesity. In this study, Biesiada et al. [30] demonstrated that the greater the maternal leptin concentration, the greater the ponderal index of the newborn. Marino-Ortega et al. [31] detected the same in a group of 118 Mexican women, observing an association between the leptin concentration of the mother and the weight/length of the newborn.

The present results confirm that BMI and gestational weight gain are determining factors for serum concentration of leptin in pregnant adolescents, as has been found in pregnant adults [4]. Consequently, body adiposity is likely the key factor contributing to serum leptin concentration in pregnant adolescents and adults as well as in their descendants. In a group of 210 mother–child dyads from Brazil, Castro et al. [32] showed that maternal leptin and adiponectin concentration correlated with the adiposity of newborn.

Likewise, the association identified in this study of the maternal leptin concentration with newborn weight and length coincides with a review done by Forhead et al. [33], which described prenatal tissue-specific effects of leptin that occur at critical periods of fetal development.

In this cohort, there was a correlation between maternal leptin concentration and length of the newborn during the first three months of life. A possible explanation for this relation is that leptin functions in synergy with other hormones, some of which have specific function in intrauterine growth. Two of those hormones, insulin and growth factor I [33], seem to be more important in the second trimester. Correlation between the leptin concentration and the weight of the newborn was significant at the different times leptin concentration at Gestation Week 28 has the greatest association with the weight of the newborn compared to the leptin concentration at Gestation Week 32 and 36. This can be understood more clearly when considering the proposal of Patenaude J et al. [34], who stated that maternal leptin concentration in the second trimester is involved in adiposity of the neonate, especially in mothers who are overweight before pregnancy.

According to the results, a positive association between the leptin concentration and birth weight of the neonate is observed. These findings are similar to those reported by Biesiada et al. [30], and Harigaya et al. [35], who observed that the higher the concentration of leptin, the greater the weight of the neonate.

Another important point to note is that the sample of pregnant adolescents who were analyzed had an average gynecological age of four years. In spite of this, their newborns had weight and length within parameters considered as normal. This is contrary to Mora-Cancino and Panduro [36,37], who reported that one of the most common complications of pregnancy in adolescents is the restriction of intrauterine growth, which results in neonates with low birth weight. Newborns in this study presented normal weight and length. This discrepancy is probably due to the excellent medical care given to these adolescents, who benefited from prenatal check-ups as of the first weeks of gestation [38]. Gestational weight gain was evaluated periodically during the pregnancy to assure the most normal evolution possible. Participants had an average weight of 51 kg at the beginning of their pregnancy and underwent an average 13 kg weight gain, representing an increase in weight within the guidelines of the IOM [22]. Finally, the current results were influenced by the fact that adolescents suffering from preeclampsia or gestational diabetes were eliminated from the study.

Adolescents in the present sample with the highest leptin concentration during Gestation Week 28, 32 and 36 were those showing the greatest pregestational BMI and the greatest weight retention at 3 months postpartum. A similar result was described by Sommer et al. [39] in a cohort of 190 Asian and 353 European women. Moreover, when comparing the Asian and European women with an elevated concentration of leptin at the onset of their pregnancy, the former group displayed greater postpartum retention of weight and subcutaneous fat.

It is important to keep in mind that excessive gestational weight gain and excessive retention of weight postpartum is one of the risk factors for developing overweight and obesity in women. In the case of adolescents, the possibility of overweight and obesity in such cases would start at a very early age. Roony and Schauberger [40] established that, 8.5 years after pregnancy, women who previously exhibited the highest postpartum weight retention were 3.5 times heavier than those without postpartum weight retention. Among the present group of adolescents, therefore, those showing the highest postpartum weight retention will likely be obese at the age of 23 or 24 years, and consequently be exposed to all the risks and metabolic complications implicit in this condition.

Serum concentration of leptin increased progressively during pregnancy. There was a close association between a higher leptin concentration and a greater weight and BMI of the adolescent. Therefore, a high concentration of leptin during pregnancy according to scientific information conditions a greater adiposity and therefore a greater resistance to the effects of leptin at the level of the central nervous system, which is manifested as an increase in gestational weight Indeed, when comparing the group with the highest versus the lowest leptin concentration in the last trimester of pregnancy, there was a weight difference of 6 kg, perhaps because leptin is a hormone that regulates weight and is synthesized in adipose tissue. Thus, the quantity of leptin circulating in the blood will be proportional to the quantity of adipocytes [32]. Castellano et al. also described similar results [17], including an increasing leptin concentration during the time of pregnancy. The same pattern can be observed in the present follow-up on postpartum weight. When comparing the pregnant adolescents with the highest (>20 ng/mL) and lowest (<20 ng/mL) leptin concentration, the postpartum weight retention was higher in the former group. Thus, adolescents having the higher leptin concentrations are exposed to a greater risk of developing overweight and obesity at an early age [29].

Finally, gestational weight gain was associated with serum leptin concentration and with intake of energy, as shown in the linear regression model. High gestational weight gain despite increased leptin levels is probably a function of both leptin resistance and other appetite regulating hormones counteracting the effect of leptin (e.g., AgRP) [41,42].

### 4.1. Strengths and Limitations of the Study

The greatest strength of this study is the methodological design, since the participants were given regular visits. In addition, they were representative of the general population of pregnant adolescents receiving attention in the hospital. Another strength is the focus on Latin women in a country with a high prevalence of obesity. In this little studied population, a correlation was found between maternal leptin concentration during pregnancy and gestational weight gain, postpartum maternal weight retention, and weight and length of the newborn.

Although some previous studies have considered these factors in pregnant adults, to our knowledge, this is the first report on such factors in pregnant adolescents. The importance of studying pregnant adolescents is that their nutritional needs are influenced by their continuing growth and development. Another advantage of the present study is the use of standard procedures for the clinical and anthropometric evaluation of the adolescents and their babies.

The main limitation is the observational design, which impedes conclusions about causality. In addition, maternal leptin levels were only determined during the pregnancy and not afterwards, and were not assessed in newborns. It must be taken into account that the results from one ethnic group, in this case Latin, are not necessarily applicable to other groups with a different complexion or eating habits.

### 4.2. Perspectives

Future studies are planned to determine whether the actions of leptin are directly involved in the growth and development of the fetus and whether other actions of this hormone may have long term consequences for the control of energetic equilibrium in adult life.

## 5. Conclusions

Adolescents having an elevated concentration of leptin during Gestation Weeks 28, 32 and 36 presented a greater gestational weight gain as well as greater postpartum weight retention during the first three months.

The study showed that leptin concentrations are associated with gestational weight gain and postpartum weight retention in a manner similar to the behavior observed in adult women.

The current results could be interpreted as the high concentration of leptin is a marker of adiposity in pregnant adolescents who gained a lot of gestational weight and therefore retained weight during the postpartum period and have high concentrations of substrate for the fetus to grow.

## Figures and Tables

**Table 1:** General characteristics of the participating adolescent mothers (*n* = 168).

Variable		Average ± SD	Range
**Mother**			
Age (years)		15.4 ± 1	12–17
Age of menarche (years)		11.4 ± 1	9–14
Gynecological age (years) ¥		4 (1–5)	0–4
Pregestational weight (kg)		51.2 ± 8	36.5–78
Maximum gestational weight (kg)		64.4 ± 8	44–102
Pregestational BMI (kg/m^2^)		21.2 ± 3	16–34
Gestational BMI (kg/m^2^) 38–40 gw		26.6 ± 3.7	20.2–39
Height (cm)		155.1 ± 4	144–165
Energy intake (Kcal) 28 gw ¥		2197 (1794–2619)	1258–2790
Energy adequacy (%) ¥		107 (88.5–128.5)	62–248
**Neonate**			
Gestational age (weeks)		39 ± 1.1	37.1–41.6
Birth weight (g)		3038 ± 380	2150–3820
Low weight ˂(−2 *Z*-score) Ŧ		10 (6)	
Adequate weight (0 *Z*-score) Ŧ		158 (94)	
Birth length (cm)		49.5 ± 2	46–53
Adequate length (0 *Z*-score) Ŧ		161 (96)	
Short length ˂(−2 *Z*-score) Ŧ		7 (4)	
Gender of newborn Ŧ			
Female		104 (62)	
Male		64 (38)	
Sociodemographic characteristics Ŧ		Frequency	Percentage
Occupation	Household duties	139	83
	Student	29	17
Marital status	Single	80	47.6
	Cohabitating/married	88	52.4
Socioeconomic level	Very low	21	13
	Low	94	57
	Medium	50	30
Educational level	Primary school (complete or incomplete)	24	14
	Middle school	103	61
	High school or above	41	24

SD: standard deviation; ¥: median (p25–p75); Ŧ: data expressed in cases (%) gw: gestation week.

**Table 2:** Correlations of maternal leptin concentrations and pregestational BMI with neonatal outcomes.

Maternal Leptin Concentration Per Gestational Weeks				Pregestational BMI
	28	32	36	
Birth weight	0.092	0.259 **	0.070	0.240 *
Weight at one month	0.113	0.196 *	0.028	0.260 **
Weight at two months	0.115	0.149	0.012	0.185 *
Weight at three months	−0.051	−0.031	−0.078	0.320 **
Length at birth	0.135	0.183 *	0.096	0.171 *
Length at one month	0.227 **	0.244 **	0.059	0.372 **
Length at two month	0.273 **	0.216 **	0.072	0.048
Length at three month	0.214 **	0.051	−0.137	0.041

All analyses were performed using a Pearson correlation. * *p* ≤ 0.050, ** *p* ≤ 0.001.

**Table 3:** Models of linear regression that explain the change in maternal gestational weight and the weight and length of the newborn.

Variable	Mean	SE	CI 95%	*p*	*R*^2^
Gestational weight gain (kg)	13.2				
Constant *		3.99	14.891, 30.891	0.001	0.192
Leptin at gw 36 (ng/mL)	23	0.03	0.100, 0.248	0.001	
Pregestational weight (kg)	51.4	0.06	−0.345, −0.072	0.003	
Adjustment of energy at gw 28 (%)	125	0.01	−0.045, −0.001	0.038	
Newborn weight (g)	3038				
Constant **		67.54	2642.9, 2009.7	0.001	0.146
Change in gestational weight (kg)	13.2	4.01	9.017, 24.858	0.001	
Leptin at gw 32 (ng/mL)	19	2.94	2.069, 13.717	0.007	
Leptin at gw 36 (ng/mL)	22	2.36	1.258, 9.592	0.049	
Newborn length (cm)	49.5				
Constant †		5.18	51.339, 71.827	0.003	0.085
Leptin at gw 32 (ng/mL)	19	0.01	0.005, 0.050	0.018	
Maternal height (cm)	155.6	0.03	0.015, 0.147	0.016	

* Adjusted for height; ** adjusted for height, energy intake adequacy percentage and pregestational weight; † adjusted for pregestational weight and gestational weight gain; CI 95%: confidence interval 95%; SE: standard error; gw: gestation week.

## Abbreviations

SD	standard deviation
BMI	body mass index
INPer	National Institute of Perinatology (Instituto Nacional de Perinatología)
IR	interquartile range
gw	gestation week
